# Could Heme Oxygenase-1 Be a New Target for Therapeutic Intervention in Malaria-Associated Acute Lung Injury/Acute Respiratory Distress Syndrome?

**DOI:** 10.3389/fcimb.2018.00161

**Published:** 2018-05-16

**Authors:** Marcelo L. M. Pereira, Claudio R. F. Marinho, Sabrina Epiphanio

**Affiliations:** ^1^Departamento de Imunologia, Instituto de Ciências Biomédicas, Universidade de São Paulo, São Paulo, Brazil; ^2^Departamento de Parasitologia, Instituto de Ciências Biomédicas, Universidade de São Paulo, São Paulo, Brazil; ^3^Departamento de Análises Clínicas e Toxicológicas, Faculdade de Ciências Farmacêuticas, Universidade de São Paulo, São Paulo, Brazil

**Keywords:** heme oxygenase, malaria, ALI/ARDS, inflammation, endothelium, heme

## Abstract

Malaria is a serious disease and was responsible for 429,000 deaths in 2015. Acute lung injury/acute respiratory distress syndrome (ALI/ARDS) is one of the main clinical complications of severe malaria; it is characterized by a high mortality rate and can even occur after antimalarial treatment when parasitemia is not detected. Rodent models of ALI/ARDS show similar clinical signs as in humans when the rodents are infected with murine *Plasmodium*. In these models, it was shown that the induction of the enzyme heme oxygenase 1 (HO-1) is protective against severe malaria complications, including cerebral malaria and ALI/ARDS. Increased lung endothelial permeability and upregulation of VEGF and other pro-inflammatory cytokines were found to be associated with malaria-associated ALI/ARDS (MA-ALI/ARDS), and both were reduced after HO-1 induction. Additionally, mice were protected against MA-ALI/ARDS after treatment with carbon monoxide- releasing molecules or with carbon monoxide, which is also released by the HO-1 activity. However, high HO-1 levels in inflammatory cells were associated with the respiratory burst of neutrophils and with an intensification of inflammation during episodes of severe malaria in humans. Here, we review the main aspects of HO-1 in malaria and ALI/ARDS, presenting the dual role of HO-1 and possibilities for therapeutic intervention by modulating this important enzyme.

## Malaria: general overview

Malaria is a serious disease caused by the *Plasmodium* parasite and transmitted by the bite of the *Anopheles* mosquito. About 212 million cases of malaria were estimated to occur in 2015, killing approximately 429,000 people in the same year, with the majority of these cases (92%) originating from sub-Saharan Africa and occurring in children under 5 years of age (70%) (WHO, 2016). In Africa and Southeast Asia, of the 3.4 billion people at risk of malaria in 2015, 1.1 billion lived in high-risk areas with more than one case of malaria reported per 1000 inhabitants (WHO, 2016). The species that cause malaria in humans are *Plasmodium falciparum, Plasmodium vivax, Plasmodium malariae, Plasmodium ovale*, and *Plasmodium knowlesi*, with the first two being responsible for the greatest number of malaria cases (WHO, 2016). A natural infection by *P. cynomolgi*, a monkey parasite, was described in a woman in Malaysia (Ta et al., 2014).

The *Plasmodium* life cycle begins when an infected female *Anopheles* mosquito bites the intermediate host, injecting sporozoites into the host's blood circulation via the mosquito's saliva. Once in the circulatory system, sporozoites reach the host's liver, where they establish an intracellular asymptomatic infection in hepatocytes (Mota and Rodriguez, 2002). In the next 7–30 days (7–15 days for *P. falciparum* and *P. vivax* and 30 days for *P. malariae*), the parasite differentiates into thousands of merozoites, which are released in the hepatic sinusoid after rupturing the hepatocytes (Prudêncio et al., 2006; Bartoloni and Zammarchi, 2012). In the blood, the merozoites will in turn infect erythrocytes. During the symptomatic phase of malaria, several clinical complications can occur, which together define what is known as severe malaria (Trampuz et al., 2003).

The species *P. falciparum* is the most virulent and predominates in Africa. On the other hand, the species *P. vivax* is less virulent and is widely distributed throughout the world (WHO, 2015). However, despite *P. vivax* being less virulent, cases of severe *P. vivax* malaria have been reported (Lacerda et al., 2012a; Baird, 2013; Rahimi et al., 2014; Im et al., 2017). In addition, *P. vivax*, as with *P. cynomolgi and P. ovale*, can form hypnozoites, the dormant parasite form, in the liver stage, and these hypnozoites can be activated weeks or months after the initial infection, causing a relapse of symptomatic blood stage infection (White and Imwong, 2012).

Severe malaria has been described as a syndrome that may affect multiple organs and is, in many ways, similar to sepsis. This makes it difficult to diagnose at an early stage because it can be confused with other febrile diseases, such as pneumonia and central nervous system infection. The major clinical complications that characterize severe malaria are severe anemia, cerebral malaria, placental malaria, acute renal failure (Kurth et al., 2017) and acute lung injury/ acute respiratory distress syndrome (ALI/ARDS) (Cowman et al., 2016).

The main pathophysiological events that occur during malaria infection are: release of pro-inflammatory cytokines, adhesion of infected erythrocytes to endothelial cells of capillaries and venules, removal of infected erythrocytes from the bloodstream by splenic macrophages and rupture of parasitized erythrocytes with consequent release of factors that activate the inflammatory response, such as glycosylphosphatidylinositol, hemozoin, DNA and precipitated uric acid derived from the parasite (Gupte, 2013; Gallego-Delgado et al., 2014; Gazzinelli et al., 2014; Wassmer and Grau, 2017). These events are associated with each other and are responsible for the major syndromes of malaria. Splenic macrophages and monocytes are mainly responsible for the release of pro-inflammatory mediators. This is because both cell types are exposed to a high number of infected erythrocytes that they phagocytose. Nevertheless, severe anemia, the most common form of severe malaria, cannot be explained solely by the phenomenon of erythrocyte removal. Moreover, a suppressive effect on erythropoiesis by pro-inflammatory cytokines has been proposed (Gazzinelli et al., 2014). In addition to macrophages and monocytes, CD4+ T cells, gamma-delta T cells and NK cells also produce pro-inflammatory cytokines, such as interleukin (IL) 12, IFN-γ (interferon gamma) and TNF (tumor necrosis factor), at an early phase of malaria infection in humans. Among these cell types, CD14+ monocytes were notable producers of TNF and other chemokines. In CD4+ T cells, IL-10 expression was predominant. The involvement of cytokines such as IL-10, IL-6, MIP-1α (macrophage inflammatory protein 1 alpha), MIP-1β (macrophage inflammatory protein 1 beta), and MCP-2 (monocyte chemoattractant protein 2) is associated with a higher likelihood of severe malaria development in humans (Stanisic et al., 2014).

Of the syndromes of severe malaria, cerebral malaria is the best characterized and understood. In humans, *P. falciparum* invades erythrocytes, causing them to express a surface protein, *P. falciparum* erythrocyte membrane protein 1 (PfEMP-1), which is responsible for erythrocyte congestion and sequestration in the brain capillaries (Horata et al., 2009; Dorovini-Zis et al., 2011). This protein causes the infected erythrocyte to adhere to the endothelial membrane via the CD36 receptor and EPCR, consequently leading to the severity of cerebral malaria (Turner et al., 2013; Almelli et al., 2014; Bernabeu et al., 2016; Brazier et al., 2017). Erythrocyte sequestration in the brain was also observed in association with axonal and myelin damage, blood-brain barrier (BBB) disruption, coma and cellular immune responses, such as fibrin–platelet thrombi, intravascular accumulation of hemozoin–containing CD45/CD68-positive monocytes, necrosis of the endothelial lining of the occluded vessel and perivascular hemorrhage (Dorovini-Zis et al., 2011; Ponsford et al., 2012). In addition, concerning severe malaria, the adhesion of infected erythrocytes in intervillous spaces is an important factor in the development of placental malaria, which can lead to impairment of fetal development, low birthweight and miscarriage (Costa et al., 2006; Umbers et al., 2011).

There are a few studies on the possible importance of malaria-associated ALI/ARDS (MA-ALI/ARDS), both *in vivo* (Corbett et al., 1989; Aitken et al., 2014; Sercundes et al., 2016) and *in vitro* (Muanza et al., 1996; Carvalho et al., 2010). Several receptors, such as ICAM-1 (intercellular adhesion molecule 1) (Carvalho et al., 2010), and CD36 (Barnwell et al., 1989; Oquendo et al., 1989; Baruch et al., 1995, 1996, 1997; Chen et al., 2000), are involved in the adhesion of parasitized red blood cells (PRBCs), leukocytes and platelets to the endothelium (Sherman et al., 2003; Wassmer et al., 2003; Kirchgatter and Del Portillo, 2005). More recently, it has been demonstrated that EPCR is involved in the cytoadhesion of PfEMP1-expressing parasites to lung endothelial cells (Avril et al., 2016), suggesting that this receptor may be involved in the pathogenesis of MA-ALI/ARDS.

The involvement of different immune cells in various organs during the appearance of the first signs of malaria reinforces the hypothesis that multiple host factors, including endothelial activation, cytoadhesion, the inflammatory response and coagulation dysregulation, are involved in the development of severe malaria syndromes, including ALI/ARDS.

## Acute lung injury/acute respiratory distress syndrome (ALI/ARDS)

In 1967, the first description of ALI/ARDS indicated that it was a syndrome characterized by severe dyspnea, tachypnea, hypoxia, loss of pulmonary compliance and the presence of diffuse alveolar infiltrates detected by chest X-ray (Ashbaugh et al., 1967). In 1994, criteria were established by the American-European Consensus to define ALI and its most severe form, ARDS (Bernard et al., 1994). However, experts met in Berlin to re-define the criteria for ALI/ARDS in 2012. According to this new definition, the term ALI was removed from use, and ARDS in humans is now defined by three subcategories, depending on the degree of hypoxemia: benign (200 mm Hg < PaO_2_/FIO_2_ ≤ 300 mmHg), moderate (100 mmHg < PaO_2_/FIO_2_ ≤ 200 mmHg) and severe (PaO_2_/FIO_2_ ≤ 100 mmHg) (Ranieri et al., 2012). In addition, other criteria were considered in the definition of ARDS, such as bilateral opacities detected by chest X-ray or by computerized tomography. The first signs of ALI/ARDS are occur either within a week after the stimulus that led to them, after a worsening of existing symptoms or after the new signs onset. Also, ARDS is defined by non-cardiogenic edema and by a positive end expiratory pressure less than 5 cm H_2_O for benign ARDS or higher than or equal to 10 cm H_2_O for severe ARDS. Finally, ARDS is defined by a corrected expired volume per minute higher than 10 L/min and/or by a low pulmonary compliance (less than 40 mL/cm of H_2_O) (Ranieri et al., 2012). Although the Berlin definition did not reach a general agreement, other authors have proposed new modifications to classify ARDS, due to low feasibility of ventilation, arterial blood gas measurements and chest radiographs, especially in developing countries (Riviello et al., 2017).

Aside from this controversy over definitions, ALI/ARDS affects more adults than children, has a high mortality rate and is characterized by dyspnea, tachypnea and hypoxemia that progress rapidly to acute respiratory failure characterized by reduced lung compliance, increased phagocytic activity and increased levels of inflammatory mediators (Monahan, 2013). The most common causes of ALI/ARDS in humans are bacterial and viral pneumonia. There are other common causes, such as sepsis derived from non-pulmonary infections, gastric aspiration and severe trauma. Other less common causes are acute pancreatitis, transfusions, adverse drug reactions and fungal or parasitic infections of the lungs (Matthay et al., 2012).

Hantavirus infection, caused by RNA virus of *Bunyaviridae* family, is characterized by flu like symptoms in the first 3 days that rapidly progress to respiratory distress, with dyspnea, lung infiltrates, pleural effusion and peribronchovascular/smooth septal thickening that are typical in ALI/ARDS of different causes (Pinto et al., 2014; de Lacerde Barbosa et al., 2017).

In animal models, there is evidence that hyperoxia-associated ALI leads to elevated levels of reactive oxygen species (ROS) that damage endothelial and lung epithelial cells, including type I pneumocytes (via cell death) and cause damage to the basement membrane (Zhang et al., 2003; Thiel et al., 2005). Furthermore, it is believed that endothelial responses are important in protection against hyperoxia-induced death due to the fact that endothelial cells constitute more than 30% of all lung cells (Crapo et al., 1982). Besides hyperoxia, there are other causes of lung endothelial damage, such as influenza virus infection, which was shown to cause platelet adhesion to the lung endothelium and thereby contribute to lung injury (Sugiyama et al., 2015). Bacterial lipopolysaccharide (LPS) is also a cause of lung endothelial cell activation and dysfunction, and its effects are mitigated by hepatocyte growth factor (HGF) (Meng et al., 2015). Recently, a murine model of hantavirus infection that mimic the lung damage in humans, was created, using immunosuppressed Syrian hamsters (Vergote et al., 2017). Histologic findings are similar to other models of ALI/ARDS: hemorrhagic alveolar edema, inflammatory cell infiltration and fibrin deposition (Vergote et al., 2017).

Endothelial damage with the consequent increase in pulmonary vascular permeability is a key factor in the pathogenesis of ALI/ARDS. This has been observed in ALI/ARDS cases associated with sepsis and pneumonia in humans, in which there is a subsequent inability to remove alveolar fluid from the alveolar epithelium (Holter et al., 1986; Constantin et al., 2007; Jabaudon et al., 2016). In murine models, it has been found that pulmonary vascular permeability is present in ALI/ARDS induced by LPS (Meng et al., 2015; Konrad et al., 2016), thrombin, histamine, TNF-alpha and VEGF. These stimuli are responsible for inducing a downstream cascade that culminates in disruption of endothelial junctions, particularly the adherens junctions, by disassembly of the VE-cadherin complex (Cerutti and Ridley, 2017).

During ALI/ARDS, the removal of extracellular fluid from the alveoli occurs due to the activity of the sodium-potassium ATPase pump in alveolar epithelial cells. In addition to the alveolar epithelium, the endothelium is also affected by the formation of spaces between the junctions of endothelial cells (Gonzales et al., 2015). As mentioned above, these spaces are formed as a result of cytoskeletal remodeling of endothelial cells induced by various inflammatory factors, such as vascular endothelial growth factor (VEGF), which is also responsible for disassembly of the VE-cadherin complex (Sukriti et al., 2014; Cerutti and Ridley, 2017). VEGF, a glycoprotein of the platelet-derived growth factor (PDGF) superfamily that is required for vasculogenesis and angiogenesis, is involved in vascular permeability and in the survival of endothelial cells (Gerber et al., 1998; Drake et al., 2000).

### Malaria-associated ALI/ARDS

MA-ALI/ARDS is a major cause of death in adults. It has a high mortality rate (80% to 100%) and can occur before, during or after anti-malarial treatment (Tran et al., 1996; Maguire et al., 2005; Kochar et al., 2009). ALI/ARDS has been diagnosed in patients suffering from malaria caused by all known species of human malaria, including *P. malariae* (Lozano et al., 1983), *P. ovale* (Haydoura et al., 2011) and *P. knowlesi* (Singh and Daneshvar, 2013). However it is more common in infections due to *P. falciparum* and *P. vivax*, with an estimated incidence of 5–25 and 1–10% respectively, in infected adults (Mohan et al., 2008; Taylor et al., 2012; Table 1). Among *P. falciparum* infected children, the incidence of ALI/ARDS varies between 7% (133 cases in 1844 infected children in Kenya) and 16% (867 cases in 5425 children from nine African countries) (Marsh et al., 1995; Dondorp et al., 2010). Pregnant women infected with *P. falciparum* are in higher risk of developing MA-ALI/ARDS. The incidence of MA-ALI/ARDS in this group is up to 29% (Taylor et al., 2012).

**Table 1:** Cases of malaria-associated pulmonary complications in humans.

**No. of cases**	**No. of deaths**	**Age(s)**	**Parasite species**	**Country**	**Respiratory signals**	**Pulmonary manifestations**	**Histology**	**References**
1	0	59	*P. vivax*	South Korea	Dyspnea	Haziness in lung XR; PaO_2_ 69,0 mmHg; FiO_2_ 1,0	No	Lee et al., 2013
200	14 (ARDS)	21–40; >60	*P. vivax* (40%); *P. falciparum* (40%); Mixed (20%)	India	Dyspnea; cough	Small airway obstruction; low O_2_ saturation; XR abnormality; ARDS	No	Nayak et al., 2011
1	0	37	*P. falciparum*	Nigeria	Dyspnea; tachypnea	PaO_2_ 49 mm Hg; bilateral bibasilar Infiltrate on XR;	No	Asiedu and Sherman, 2000
1	1	60	Unknown	India	Unknown (patient was found dead)	Edematous lungs	PRBC with hemozoin; alveolar exudate; focal pleural fibrosis; lymphocytic infiltrate; septal vessels with PRBC	Menezes et al., 2010
2	1	40 and 50	*P. falciparum*	India	Dyspnea	Diffuse alveolar hemorrhages; bilateral diffuse infiltrates on XR; PaO_2_ 60 and 78 mmHg; FiO_2_ 1 and 0,6	No	Saigal et al., 2014
1	1	20	*P. vivax*	India	Dyspnea	ARDS	Focal pulmonary edema; alveolar capillary congestion; diffuse alveolar damage; hyaline membrane formation; inflammatory infiltrate	Valecha et al., 2009
100	0	18–44	*P. vivax* (50%); *P. falciparum* (50%)	Indonesia	Cough	RF: > 24 breaths/min; reduced gas transfer	No	Anstey et al., 2007
10	10	17–34	*P. falciparum*	Mozambique	Dyspnea	Not described	PRBC in lung microvasculature; lung edema; hemorrhage and congestion	Castillo et al., 2013
1	0	28	*P. vivax*	India	Dyspnea	RF: 44 breaths/min.; PaO_2_/FiO_2_:100; bilateral alveolar opacities on XR; respiratory alkalosis	No	Agarwal et al., 2007
1	0	38	*P. falciparum*	Brazil	Dyspnea	Bilateral pulmonary infiltrates; interlobular septal thickening; bilateral pleural effusion	No	Marchiori et al., 2013
19	19	1–88	*P. vivax*	Brazil	Not described	Lung edema; ARDS	Alveolar edema; interstitial infiltrate; congestion; hyaline membrane	Lacerda et al., 2012b
204	5 (ARDS)	>11	*P. falciparum*	India	Cough; dyspnea; tachypnea;	RF: greater than 30 breaths /min; XR abnormal; pulmonary infiltrates	No	Maria et al., 2015
120	14	15–71	*P. falciparum*	Thailand	Not described	Lung edema; ARDS (PaO_2_ < 60 mm Hg; FiO_2_ > 60 percent)	No	Aursudkij et al., 1998
26	0	18–72	*P. falciparum*; *P. vivax; P. ovale*	Australia	Cough	Airflow obstruction; worsening of gas transfer; increased pulmonary phagocytic activity	No	Anstey et al., 2002
100	100	0, 5–13	*P. falciparum*	Malawi	Not described	RF: 42–55 breaths/min;	Pulmonary edema; alveolar hemorrhage; alveolar fibrin; microthrombi; PRBC in alveolar capillaries; hemozoin deposits	Milner et al., 2013
800	95	0, 5–15	*P. falciparum*	Burkina Faso	Not described	Respiratory distress; pulmonary edema	No	Modiano et al., 1998
922	11	≥18	*P. vivax, P. falciparum*	India	Not described	Respiratory distress; pulmonary edema; rising of respiratory rate	No	Saravu et al., 2014
48	24	13–82	*P. vivax, P. falciparum*	India	Dyspnea	PaO_2_/FiO_2_ < 200 mm Hg (in 25 patients;	Edematous lungs, congestion of alveolar capillaries with mononuclear cells (one patient)	Londhe et al., 2014
103	103	Children	*P. falciparum*	Malawi	Not described	Not described	PRBCs in vasculature; intravascular accumulation of monocytes/macrophages; pulmonary edema; capillary microthrombi	Milner et al., 2014
3	0	15–42	*P. vivax*	India	Dyspnea	RF: 32–48 breaths/min; PaO_2_/FiO_2_ < 200 mm Hg; hemoptysis	No	Sarkar et al., 2010
100	1	18–50	*P. vivax*	India	Dyspnea; tachypnea; dry cough; runny nose; sore throat	ARDS	No	Muley et al., 2014
1	0	73	*P. knowlesi*	Myanmar/ Thailand	Respiratory distress; Tachypnea	Respiratory distress RF: 18–20 breaths/min; interstitial opacities; pleural effusion; congestion; bilateral basal infiltrations	No	Seilmaier et al., 2014
3	3	16–28	*P. falciparum*	Brazil	Dyspnea; respiratory failure	Lung congestion on XR; PaO_2_/FiO_2_ < 200 mm Hg; edema	No	Fernandes et al., 2010

Respiratory symptoms such as cough, respiratory rate increase and a decrease in forced midexpiratory flow can be present even in uncomplicated malaria cases. However, most patients suffering from severe malaria that develop ALI/ARDS are characterized by the presence of pulmonary vascular occlusions and impaired alveolar capillary membrane function (Maguire et al., 2005; Anstey et al., 2007). Additionally, in patients with *P. vivax* malaria, a reduced pulmonary capillary vascular volume of hemoglobin-containing cells was suggested to occur due to the sequestration of infected erythrocytes within the pulmonary vasculature (Anstey et al., 2007).

It is known that the presence of intravascular fluid in the lungs due to increased alveolar permeability is the key pathophysiological mechanism of MA-ALI/ARDS (Mohan et al., 2008). Multiple factors are possibly involved in this increased permeability, such as increased levels of pro-inflammatory cytokines such as TNF-α, IL-1, IL-6, and IL-8. These factors may also include the endovascular occlusions associated with the accumulation of erythrocytes with reduced deformability, leukocytes and infected erythrocytes as well as the endothelial injury caused by adhesion of infected erythrocytes (Mohan et al., 2008). As previously mentioned, a number of receptors participate in the adhesion of infected erythrocytes, such as CD36, ICAM-1 and EPCR (Carvalho et al., 2010; Avril et al., 2013, 2016; De las Salas et al., 2013). The *P. falciparum* PfEMP1 protein is known to interact with ICAM-1, an endothelial adhesion molecule that participates in leukocyte transmigration across endothelial cells, in a process that involves the activation of a signaling cascade that leads to disruption of VE-cadherin junctions (Sherman et al., 2003; Cerutti and Ridley, 2017). Additionally, the adhesion of *P. falciparum* parasitized erythrocytes to CD36 (Sherman et al., 2003) initiates a pro-inflammatory cascade in mononuclear cells through the association of CD36 with Lyn and Fyn, which are members of the Src phosphotyrosine kinase family (Moore et al., 2002). Fyn was shown to be activated in a TLR4 mediated cascade, culminating in the disruption of VE-cadherin junctions and a consequent increase in permeability (Gong et al., 2008). More recently, it was shown that knockdown of CD36 and Fyn reduced lung endothelial barrier dysfunction in a murine model of malaria (Anidi et al., 2013). Expression of VEGF was found to be increased in a murine model and was involved in the increase in vascular permeability observed in MA-ALI/ARDS (Epiphanio et al., 2010). This syndrome may also be associated with other factors, such as heart failure or tachypnea associated with cerebral malaria (Good et al., 2005). Thus, many factors are associated with endothelial permeability and are essential mechanisms of MA-ALI/ARDS. However, the biomarkers that are critical to the development of ALI/ARDS are not yet well defined (Janz and Ware, 2013). Therefore, the study of pro-inflammatory biomarkers, particularly cytokines or proteins related to cellular stress (such as heme oxygenase), is of central importance.

### Malaria-associated ALI/ARDS models

It has been shown that the pathogenesis of MA-ALI/ARDS is multifactorial and that PRBC endothelium sequestration, inflammation and hemostasis are all involved in the pathogenesis of severe malaria, especially cerebral malaria (van der Heyde et al., 2006), are also relevant in the development of MA-ALI/ARDS.

Murine models are an important connection between patients from an endemic zone and the research laboratory. Emerging hypotheses in human studies can be tested in animal models, which provide valuable information on the pathogenesis of a disease. Additionally, different murine models have been developed to study MA-ALI/ARDS and show aspects similar to human ALI/ARDS cases (Table 2).

**Table 2:** Mouse models of ALI/ARDS.

**Mouse strain**	**Parasite species**	**Organs affected**	**Lung findings and inflammatory response**	**Interventions**	**References**
129P2Sv/ev	*P. berghei* ANKA	Brain and lungs	Lung edema; accumulation of T cells and neutrophils	IFN-γR1-KO mice do not develop lung edema	Belnoue et al., 2008
BALB/c	*P. yoelii* 17XL; *P. yoelii* 17XNL	Brain, spleen, kidneys, liver, lungs	Lung edema; infiltration of mononuclear inflammatory cells; PMN leukocytes in the alveolar wall	No lung pathology in *P. yoelii* 17XNL	Fu et al., 2012
C3H/z	*P. berghei*	Lungs	Lung edema, alveolar leakage of serum proteins, granular precipitates in the alveoli	None	Weiss and Kubat, 1983
C57BL/6	*P. berghei* K173	Lungs	Lung edema; leukocyte infiltration into the interstitium; thickened alveolar septa; congested capillaries; neutrophils, monocytes, T cells increased	No lung pathology with Phenylhydrazine	Hee et al., 2011
C57BL/6	*P. berghei* NK65; *P. berghei* ANKA	Brain (ANKA); lungs (NK65 and ANKA)	Increased lung weight; swollen lungs; interstitial edema; leukocyte infiltrations; hyaline membrane; edema; hemorrhage; TNF-alpha, IL-10, CXCL10, CXCL11 increase (NK65 only)	CD8+ T cell depletion and dexamethasone inhibited lung pathology;	Van den Steen et al., 2010
C57BL/6	*P. berghei* ANKA	Brain; lung; hearth; liver; kidneys;	Alveolar collapse; neutrophil infiltration; interstitial edema; IFN-gamma, TNF-alpha, CXCL1 increase	–	Souza et al., 2013
C57BL/6	*P. berghei* ANKA	Brain; lung	Alveolar-capillary membrane barrier disruption; interstitial pulmonary inflammation; inflammatory cells in the alveolar septae; IFN-gamma, TNF-alpha, IL-10, IL-6, IL-8, MIP-2 increase	Less lung pathology in CD36 KO	Lovegrove et al., 2008
C57BL/6	*P. berghei* GFP-luciferase	Brain; lungs	Sequestration of parasite in lung	Reduced sequestration in CD36 KO	Franke-Fayard et al., 2005
C57BL/6	*P. berghei* ANKA	Brain; lungs; kidney; heart	Increased vascular permeability	IL-12 KO, TNFR1 KO, IFN-gamma KO and ICAM-1 KO with less lung permeability	van der Heyde et al., 2001
C57BL/6	*P. berghei* ANKA	Brain; lungs; spleen	Increased vascular permeability, TNF levels and cell sequestration	CD40 KO and CD40L KO mice with less lung permeability but more TNF	Piguet et al., 2001
C57BL/6	*P. berghei* ANKA	Brain; lungs	Increased TNF levels, PMN cell sequestration and macrophage numbers	ICAM-1 KO with less macrophage numbers in lung	Favre et al., 1999
C57Bl/6	*P. berghei* NK65	Lungs	Alveolar edema; hemozoin increase; cytoadherence of PRBCs; increased levels of CXCL10, CXCL1, CCL2, IL-1 beta, IL-4, IL-10, TNF, TGF-beta, HO-1, and VEGF	Hemozoin injection mimics the pathology of the infected mice	Deroost et al., 2013
C57Bl/6	*P. berghei* ANKA	Brain; liver; spleen; lungs	Increase of IFN-gamma receptor in CD4+ and CD8+ T cells;	Less CCR5 and CXCR3 in CD4+ T cells of IFN KO mice	Villegas-Mendez et al., 2011
C57BL/6	*P. berghei* ANKA; *P. berghei* ANKA Hb^SAD^	Brain; liver; lungs	Severe lung injury	Less lung injury with *P. berghei* ANKA Hb^SAD^, except when in Hmox^+/−^ or Nrf2^+/−^	Ferreira et al., 2011
C57BL/6J	*P. berghei* ANKA	Brain; lungs	Thickened alveolar septae; alveolar edema; inflammatory cell infiltration; increased number of parasites and ROS	No lung pathology in CD36 KO; Fyn KO with reduced lung endothelial permeability	Anidi et al., 2013
CBA/J	*P. berghei* ANKA	Brain; liver; lungs	Lung edema; adhesion of hemozoin-containing monocytes and neutrophils; septal pneumonitis; monocyte infiltrates	–	Carvalho et al., 2000
DBA/2	*P. berghei* ANKA	Lungs	Dyspnea; neutrophils, lymphocytes, monocytes and macrophages in pleural fluid; edema	Increased serum VEGF and spleen VEGF mRNA in ALI/ARDS mice; splenectomised, VEGF neutralized and CO treated mice are protected against ALI/ARDS	Epiphanio et al., 2010
DBA/2	*P. berghei* ANKA	Lungs	Pleural effusion; alveolar edema; hemorrhage; neutrophil infiltration; destruction of the alveolar septa; lung opacity on XR; increased vascular permeability	None	Ortolan et al., 2014
DBA/2	*P. berghei* ANKA	Lungs	Pleural effusion; red swollen lungs; edema, hemorrhage; thickened septa; congested capillaries; leukocyte infiltrate	None	Aitken et al., 2014
DBA/2	*P. berghei* ANKA	Lungs	Dyspnea; respiratory insufficiency; pulmonary exudate; vascular congestion with PRBCs	ALF492-treated mice with reduced lung pathology and reduced VEGF in serum	Pena et al., 2012
DBA/2	*P. berghei* ANKA	Lungs	Inflammatory infiltrate with numerous neutrophils; increased CXCL-1 and 2, ROS, MPO, NET formation	Depletion of neutrophils, NETs (Pulmozyme) and treatment with CXCR4 antagonist protected against ALI/ARDS	Sercundes et al., 2016
ICR	*P. berghei* ANKA	Brain; liver; spleen; kidneys; lungs	Interalveolar PRBC congestion; hyaline membrane; lung inflammation	WSX-1Fc reduced PRBC congestion; IL-27 or WSX-1Fc prevented hyaline membrane formation	Fazalul Rahiman et al., 2013
Swiss	*P. berghei* ANKA	Lungs; liver; spleen	Increased lung weight; hemorrhage; increased macrophages and lymphocytes;	Splenectomy reduced the lung weight	Moore et al., 2008

The DBA/2 mouse strain develops ALI/ARDS when infected with the parasite *P. berghei* ANKA (Epiphanio et al., 2010; Ortolan et al., 2014). Around 50% of the mice from this strain that die between the 7th and 12th days after infection have dyspnea, hypoxemia, reduced respiratory rate and lung opacification identified by X-ray analysis (Epiphanio et al., 2010; Ortolan et al., 2014). Post-mortem studies revealed that these mice had pleural effusions containing neutrophils, lymphocytes, monocytes and macrophages (Aitken et al., 2014; Ortolan et al., 2014; Sercundes et al., 2016). When infected with *P. berghei* NK65, C57BL/6 mice also develop severe malaria that is characterized by cellular infiltration, edema in lung interstitial tissue, alveolar edema and hyaline membrane formation, which is typical of ALI/ARDS. In this model, approximately 90% of infected mice die from ALI/ARDS (Van den Steen et al., 2010). In these two models, the mice develop MA-ALI/ARDS without developing signs of cerebral malaria, which make them useful for studying this syndrome. Furthermore, VEGF has been identified as being a critical mediator of increased pulmonary vascular permeability, a hallmark of ALI/ARDS (Epiphanio et al., 2010; Pena et al., 2012). In addition to VEGF, cytokines and chemokines, such as TNF, IL-10 (interleukin 10), IL-1β (interleukin 1 beta), IL-6, IL-4, INF-γ, chemokine (C-C motif) ligand 2 (CCL-2), chemokine (C-X-C motif) ligands (CXC) CXCL1, CXCL2, CXCL10, CXCL11, and myeloperoxidase (MPO), were shown to be increased during ALI/ARDS in other studies (Favre et al., 1999; Piguet et al., 2001; Lovegrove et al., 2008; Van den Steen et al., 2010; Deroost et al., 2013; Souza et al., 2013; Sercundes et al., 2016). Recently, the formation of *Plasmodium*-induced neutrophil extracellular traps (NETs) has been found to contribute to MA-ALI/ARDS pathogenesis (Sercundes et al., 2016). In addition to causing inflammatory factor induction, malaria infection also leads to the release of ROS and free heme from infected erythrocytes during the blood stage (Ferreira et al., 2008). These compounds are harmful to the host, which reacts by increasing the expression of heme oxygenase (HO-1), a heme catabolizing enzyme (Ferreira et al., 2008).

### The heme oxygenase system

The heme oxygenase system has been intensively studied due to its regulatory properties in both physiological and pathological processes. Heme oxygenase has two isoforms: HO-1, which is inducible and heme oxygenase-2 (HO-2), which is constitutive. The enzymatic activities of both isoforms are identical. HO-2 possibly regulates normal physiological cellular functions, whereas HO-1 is expressed in all cells and is highly inducible by a variety of stimuli. HO-1, encoded by the *hmox-1* gene, is considered protective due to its anti-inflammatory, anti-apoptotic and anti-proliferative actions in different cell types, including endothelial cells (Soares et al., 1998). HO-1 is expressed in all cells at low levels, but is rapidly inducible by various stimuli, including heme (Agarwal and Bolisetty, 2013). This enzyme participates in the degradation of free heme, derived from hemoglobin and myoglobin, into equimolar amounts of CO, iron and biliverdin. Biliverdin is subsequently reduced to bilirubin by biliverdin reductase. HO-1 plays a protective role in various organs by modulating tissue responses to injuries, including the lung injury associated with hyperoxia as well as pulmonary hypertension and pulmonary fibrosis (Otterbein et al., 1999; Christou et al., 2000; Tsuburai et al., 2002).

HO-1 provides cellular protection through the degradation of free heme, because this action has pro-oxidant effects (Balla et al., 1991; Tracz et al., 2007). Additionally, the HO-1 reaction products also contribute to the protective response. In response to oxidative stress, cells change their gene expression to activate protective genes. The transcription factor Nrf2 and its target genes (involved in antioxidative cell protection) are important in protecting against ROS and cell damage. (Stocker et al., 1987; Nakagami et al., 1993; Motterlini and Otterbein, 2010).

Several drugs are known to modulate the expression of HO-1. A compound called desoxyrhapontigenin decreases ROS and peroxynitrite production and induces HO-1 via activation of Nrf2. This drug also leads to an improvement in pulmonary inflammation induced by LPS in ICR (imprinting control region) mice (Joo Choi et al., 2014). Besides desoxyrhapontigenin, other compounds such as statins, hemin, curcumin, quercetin, carnosol and cobalt (inducers) and tin and zinc protoporphyrins (inhibitors) are also known modulators of HO-1. (Pamplona et al., 2007; Fei et al., 2012; Zhou et al., 2013; Luo et al., 2014; Chi et al., 2016; Pereira et al., 2016; Yu et al., 2016; Immenschuh et al., 2017). Moreover, carbon monoxide (CO), one of the end products of the HO-1 reaction, shows the same anti-inflammatory, anti-apoptotic, and anti-proliferative effects as HO-1. For this reason, CO-releasing molecules (CO-RMs) (lipid-soluble CORM-2 [Ru(CO)3Cl2]2, and water-soluble CORM-3 [Ru(CO)3Cl2 (H2NCH2CO)2], CORM-401 and ALF499) (Pena et al., 2012; Choi et al., 2015; Hettiarachchi et al., 2017; Inoue et al., 2017; Sun et al., 2017) have been broadly used. Hence, modulating HO-1 expression by pharmacologically targeting this enzyme has promise as a therapeutic application in humans and could be used to inhibit pulmonary inflammation.

### The modulation of HO-1 in ALI/ARDS

It has been shown that the inhibition of HO-1 led to a worsening of ALI/ARDS signs in rats after ischemia-reperfusion (I/R) of the lower limbs (Boutros et al., 2005). On the other hand, cobalt protoporphyrin and hemin, both inducers of HO-1, were shown to be protective against ALI/ARDS (Yin et al., 2011; Pereira et al., 2016; Wang et al., 2017). HO-1 is also involved in endotoxemia, and the induction of HO-1 expression led to a reduction of LPS-induced ALI/ARDS in rats (Otterbein et al., 1995).

Likewise, in a sepsis-induced ALI/ARDS murine model it has been shown that hemin inhibits NLRP3 inflammasome activation through the action of HO-1 (Luo et al., 2014). The NLRP3 inflammasome regulates the maturation of the pro-inflammatory cytokines IL-1β and IL-18 and can be activated by various stimulating factors such as bacteria, viruses, fungi and DAMPS (danger associated molecular patterns) (Schroder and Tschopp, 2010; Anand et al., 2011). Deregulated activation of NLRP3 is present in sepsis-associated ALI/ARDS in mice, and in the absence of NLRP3 activation there is a reduction in pro-inflammatory cytokine and neutrophil levels in bronchoalveolar lavage fluid (Grailer et al., 2014).

Additionally, hemin treatment reduces the levels of oxidative stress markers in lung tissue and reduces the severity of ALI/ARDS in treated mice compared to untreated mice or mice treated with the HO-1 inhibitor zinc protoporphyrin IX (ZnPPIX) (Luo et al., 2014). However, it was recently shown that ZnPPIX has a deleterious effect in a cecal ligation and puncture (CLP) mouse model. In this study, it was shown that the antimalarial drug artesunate induced HO-1 gene expression, and treated mice exhibited less pronounced sepsis mortality, lung injury and neutrophil infiltration (Cao et al., 2016). Also, in a recent study, cobalt protoporphyrin IX was shown induce HO-1 in human cells and in mice infected by human respiratory syncytial virus (hRSV) (Espinoza et al., 2017). The hRSV virus is the main cause of lower respiratory tract illness in children up to five years old worldwide (Rudan et al., 2013). The induction of HO-1 in human respiratory tract epithelial cells led to a reduction of hRSV replication. Additionally, the levels of HO-1 were increased in infected dendritic cells *in vitro* and in HO-1 inducer-treated mice, which were protected due to a reduction in virus replication and to a decrease of neutrophil infiltration and inflammation in their lungs (Espinoza et al., 2017).

In another study it was observed that HO-1 deficient mice had decreased survival to influenza virus infection, increased levels of lung inflammation and also had impaired production of antibodies following influenza vaccination when compared with wild type mice (Cummins et al., 2012). Also, humans with SNPs in both HO-1 and HO-2, expressing lower levels of them, showed to have lower production of antibodies after influenza vaccination (Cummins et al., 2012). These results reinforce the hypothesis that the observed increased expression of HO-1 in patients may be due to host efforts to reverse the phenotype of ALI/ARDS.

To study the role of HO-1 in ALI/ARDS, chemical inhibitors, such as ZnPPIX, or *hmox-1* gene deletion (HO-1-KO) can be used in murine models. However, the use of metalloporphyrins has been shown to have adverse effects, which may interfere with the results of studies on HO-1 (Grundemar and Ny, 1997). Additionally, HO-1-KO tests have the following problems: partial lethality during prenatal development; infertility; smaller size of the HO-1-KO mice relative to wild-type mice; development of microcytic normochromic anemia in HO-1-KO animals; iron deposition in organs, such as the liver and kidneys, which can affect the results of the experiments (Agarwal and Nick, 2000; Gozzelino et al., 2010).

To overcome these problems, it was found that specific inhibition of pulmonary HO-1 through interfering RNA (siRNA) makes it possible to study the effect of post-natal silencing of HO-1 in a lung-targeted manner. To achieve this, Zhang et al. designed specific lentiviral constructs for certain lung cells (Zhang et al., 2013). In animal models, hyperoxia leads to the presence of high amounts of ROS, which damage lung endothelial and epithelial cells, including type I pneumocytes (via cell death), and cause damage to the basement membrane (Zhang et al., 2003; Thiel et al., 2005). When lentiviruses were used to silence HO-1 expression in lung endothelial cells in a model of hyperoxia-induced acute lung injury (HALI), increases in inflammatory cytokines (IL-1β, IL-6 and TNF-α), apoptosis (caspase 3-mediated) and a decrease in autophagy were observed (Zhang et al., 2013). Finally, it was concluded that the HO-1-knockdown in endothelial cells was just as bad as in the whole lung in a murine model of hyperoxia, since there was a reduction in survival compared to the controls in both cases (Zhang et al., 2013). This emphasizes the importance of endothelial cells in controlling the inflammatory response via HO-1 expression.

### The dual role of HO-1 in malaria

The role of HO-1 in malaria has long attracted interest among the scientific community and clinicians (Pamplona et al., 2007; Walther et al., 2012; Pereira et al., 2016). There is a number of studies in which HO-1 was observed to be important in malaria infection in humans and in murine models, because its expression was increased during the infection or because its modulation had an effect on the development of the disease (Table 3).

**Table 3:** Studies on the role of HO-1 in malaria.

**Parasite species**	**Host**	**Malaria complications**	**HO-1 expression**	**HO-1 modulation**	**Main effects**	**References**
*P. berghei*	C57BL/6	Brain, lung and kidney damage	Plasma; kidney; brain; lung; CXCL10 depletion downregulated HO-1	Heme; CoPP IX; ZnPP IX; (mouse endothelial cell line)	Heme and CoPP IX upregulated HO-1; ZnPP IX downregulated HO-1 (mRNA and protein)	Liu et al., 2012
*P. berghei; P. yoelii*	C57BL/6; Balb/c; *hmox-1* KO	No complications (liver stage malaria)	Liver; expression increases with malaria infection	Adenovirus expressing HO-1; siRNA to deplete HO-1	HO-1induction is required for the infection establishment; overexpression of HO-1 increases *P. berghei* liver infection.	Epiphanio et al., 2008
*P. berghei* ANKA	C57BL/6 and Balb/c	Cerebral malaria (C57BL/6)	Brain	CoPP IX; ZnPP IX; CO	HO-1 induction or CO suppresses ECM; ZnPP IX and HO-1 KO led to ECM in Balb/c mice	Pamplona et al., 2007
*P. berghei ANKA*	DBA/2	ALI/ARDS	Higher HO-1 expression in lung and serum during ALI/ARDS	Hemin	HO-1 induction protected the mice against ALI/ARDS	Pereira et al., 2016
*P. falciparum*	Human	Cerebral malaria	Brain (areas of bleeding)	No	No modulation	Schluesener et al., 2001
*P. falciparum*	Human	Cerebral malaria	Brain; lung (monocytes and alveolar macrophages); liver	No	No modulation	Clark et al., 2003
*P. falciparum*	Human	Cerebral malaria	Higher frequency of homozygotes for short repeat alleles in the HO-1 gene in cerebral malaria than in uncomplicated malaria	No	No modulation	Takeda et al., 2005
*P. falciparum*	Human	Severe malaria (5%)	Plasma; whole-blood and blood monocytes increased HO-1 in subjects with acute malaria	No	No modulation	Cunnington et al., 2012
*P. falciparum*	Human	Cerebral malaria	One *hmox-1* haplotype was associated with increased HO-1 expression in the blood and with the risk of developing cerebral malaria	No	No modulation	Sambo et al., 2010
*P. falciparum*	Human	Severe malaria	HO-1 blood levels and neutrophils showed higher HO-1 expression; Higher HO-1 expressing short variant alleles associated with severe malaria	Hemin; SnPP	Hemin increased HO-1 expression in isolated neutrophils and increased oxidative burst. The latter was reversed by HO-1 inhibition (SnPP)	Walther et al., 2012
*P. vivax* (>90%); *P. falciparum*	Human	Malaria severity evaluated through liver inflammation markers	Higher HO-1 expression in symptomatic malaria; short form of the *hmox-1* gene (more expression) associated with less susceptibility to malaria	No	No modulation	Mendonça et al., 2012
*Plasmodium chabaudi chabaudi*	Balb/c *hmox-1* KO and DBA/2	Hepatic failure (not observed in Balb/c wild type)	Liver (DBA/2)	Adenovirus expressing HO-1	Adenovirus-treated DBA/2 suppressed liver damage	Seixas et al., 2009

As in the cases of hyperoxia, endotoxemia and I/R, malaria infection also leads to the release of ROS and free heme, both of which are harmful to the host's endothelial cells. In addition, heme influences neutrophil activation, leading to a respiratory burst, chemotaxis and formation of NETs (Immenschuh et al., 2017). However, HO-1 in turn catabolizes free heme into iron, biliverdin and CO, which are less toxic to the cells. When exposed to free heme, host cells increase expression of HO-1 (Ferreira et al., 2008).

However, although some of those studies conclude that HO-1 plays a protective role (Pamplona et al., 2007; Seixas et al., 2009), it is known that expression of this inducible enzyme is not always beneficial. At the beginning of the malaria infection, that is, in the asymptomatic phase (hepatic stage of the disease), HO-1 expression is harmful, since it reduces inflammation and consequently provides a favorable environment for the development and multiplication of parasites (Epiphanio et al., 2008).

In addition, a study performed in Gambian children (Walther et al., 2012) showed that high levels of HO-1 were associated with severe malaria. The authors also observed an increase in HMOX1 mRNA expression in inflammatory cells. Surprisingly, they induced HO-1 expression in neutrophils using hemin, which increases the oxidative burst (Walther et al., 2012), suggesting that serious damage to endothelial cells is the mechanism by which HO-1 contributes to ALI/ARDS. On the other hand, the increase in HO-1 induced by chronic hemolysis induces an accumulation of immature granulocytes in the blood, leading to a reduction in the oxidative burst (Cunnington et al., 2011). This decrease in respiratory burst could favor new bacterial infections (Cunnington et al., 2011), predisposing patients with malaria to co-infections, which is also a potential mechanism that could facilitate ALI/ARDS (Lacerda et al., 2012b; Mueller et al., 2012).

Recently, it has been found that *Mycobacterium tuberculosis* induced the HO-1 overexpression in macrophages from humans and mice, in a mechanism that was dependent on the presence of ROS. HO-1 increased levels were also observed during HIV coinfection, which were directly proportional to the viral load and to the severity of tuberculosis. It was also concluded that HO-1 is useful as a biomarker for both tuberculosis infection and treatment (Rockwood et al., 2017). Additionally, the dependence of HO-1 levels on ROS reinforces the possibility that the HO-1 increase constitutes an effort to revert the exacerbated inflammatory response and minimizes the damage caused by ROS. Thus, despite being increased during the worsening phase of tuberculosis and malaria, the role of HO-1 in these diseases seems to be beneficial. However, the stage of malaria infection seems to be important in defining the role of HO-1. At the beginning of a malarial infection at the liver stage, an increase in the HO-1 levels might be deleterious because it lowers the inflammatory response, which is important to reduce the parasite numbers at this stage. Nevertheless, in posterior stages of infection (blood stage), characterized by the accumulation of free heme as a result of parasitized erythrocyte burst and inflammatory response, HO-1 increase would be more beneficial as it would prevent the toxic effects of heme and reduce an exacerbated inflammation.

It has been observed that MA-ALI/ARDS in humans frequently occurs at late stages of malaria infection and even after the completion of the malaria treatment (Tong et al., 1972; Taylor et al., 2006). Additionally, it has been hypothesized that the accumulation of hemozoin in lung, which stays in tissue even after the parasite clearance, plays an important role in MA-ALI/ARDS. As it has been shown, hemozoin is a potent inducer of the inflammatory response and is correlated with MA-ALI/ARDS (Deroost et al., 2013). Therefore, HO-1 might be a target to prevent MA-ALI/ARDS, avoiding the oxidative burst and deleterious effects of an exacerbated and harmful inflammatory response.

Besides the previous observations in humans, different studies have shown the effects of modulating HO-1 in different infection models (Table 4). These studies have also demonstrated that inducers of HO-1, such as hemin and cobalt protoporphyrin IX (CoPPIX), protected mice infected with malaria or suffering from other diseases, such as polymicrobial sepsis, from ALI/ARDS (Pamplona et al., 2007; Fei et al., 2012; Luo et al., 2014). Treatment with HO-1 inhibitors, such as ZnPPIX or tin-protoporphyrin, led to a worsening of ALI/ARDS signs in cases of sepsis and hyperoxia, but had no effect in experimental cerebral malaria (ECM) (Pamplona et al., 2007; Siner et al., 2007; Ballinger et al., 2012; Fei et al., 2012). In C57BL/6 mice, the induction of HO-1 suppressed the pathogenesis of ECM, preventing the breakdown of the blood brain barrier and reducing neuroinflammation (Pamplona et al., 2007). However, chemically inhibiting HO-1 with ZnPPIX had no effect on pathogenesis in C57BL/6 mice. Nevertheless, it was observed that HO-1-KO Balb/c mice died due to this form of severe malaria, while the wild-type strain did not develop ECM. The same result was observed in Balb/c mice treated with ZnPPIX (Pamplona et al., 2007). Furthermore, it was shown that the induction of HO-1 expression by inoculation of mice with a recombinant adenovirus containing the HO-1 gene protected them against malaria-associated hepatic failure (Seixas et al., 2009). We previously observed that DBA/2 ALI/ARDS-developing mice infected by *Plasmodium berghei* have increased levels of the HO-1 protein in their lungs and serum, and HO-1 induction improved respiratory parameters, reduced inflammatory cytokines and protected the alveolar capillary barrier in these mice (Pereira et al., 2016). On the other hand, in a study performed in humans, it was found that the variant of the HO-1 promoter (hmox1) with the shortest repeating (*GT*)_*n*_ (*n* < 27), was associated with higher HO-1 expression in peripheral blood leukocytes compared to the variant with the longest repetition (*n* > 32) (Walther et al., 2012). High HO-1 expression led to higher levels of HO-1, and those elevated levels were associated with severe malaria complications, including ALI/ARDS (Takeda et al., 2005; Sambo et al., 2010; Cunnington et al., 2012; Walther et al., 2012). However, in a study in Brazil, the opposite was observed. Asymptomatic malaria was more frequent in individuals carrying the short form of the HO-1 allele (Mendonça et al., 2012). Importantly, it was shown that the development of MA-ALI/ARDS is suppressed by administration of exogenous CO, and high VEGF levels are associated with the development of this syndrome (Epiphanio et al., 2010). In addition, pulmonary endothelial permeability levels were increased in this murine model, which also exhibited alveolar edema and pleural effusion (Epiphanio et al., 2010; Ortolan et al., 2014). The induction of HO-1 by hemin treatment led to not only a reduction in the mortality due to MA-ALI/ARDS, but also to a decrease in VEGF protein levels in serum and to protection of the endothelial cells against apoptosis (Pereira et al., 2016). Therefore, understanding the mechanisms of endothelial survival factors, such as HO-1 and VEGF, may have important therapeutic implications. Our data have shown that there might be a mechanism of regulation between HO-1 and VEGF. Consequently, it is important to determine the mechanism by which HO-1 has a protective role in MA-ALI/ARDS.

**Table 4:** Modulation of HO-1 in different disease experimental models.

**Species and Strain**	**Cell line**	**HO-1 inducer**	**HO-1 inhibitor**	**HO-1 localization**	**Effect**	**References**
Human	Isolated monocytes	Hemin	ZnPPIX	Monocytes	More HO-1 leads to less apoptosis in monocytes	Lang et al., 2005
Human	Isolated neutrophils	Hemin	SnPP	Neutrophils	More HO-1 associated with severe malaria	Walther et al., 2012
Kunming mouse	–	Artesunate	ZnPPIX	Lungs	More HO-1 leads to ALI induced by CLP	Cao et al., 2016
Mouse C57BL/6	–	A toxin (enteritis)	SnPP	F4/80 macrophages	More HO-1 leads to more CX3CR1 and less enteritis	Inui et al., 2011
Mouse Balb/c	Isolated peritoneal cells	Hemin	–	Peritoneal and pancreatic macrophages	More HO-1 leads to less acute pancreatitis and ALI	Nakamichi et al., 2005
Mouse C57BL/6	CRL-2581 - murine endothelial cells	Heme and COPPIX	ZnPPIX	Kidneys, brain and lungs	*P. berghei* infection leads to more HO-1 in ECM	Liu et al., 2012
Mouse C57BL/6	–	Hemin	ZnPPIX	Lungs	More HO-1 leads to less ALI/ARDS induced by sepsis	Luo et al., 2014
Mouse C57BL/6	HUVECs and MECs	CoPPIX	ZnPPIX	HUVECs and MECs	More HO-1 leads to less complement mediated vascular endothelium injury	Kinderlerer et al., 2009
Mouse Balb/c	RAW 264.7 cells	CoPPIX	ZnPPIX	–	More HO-1 leads to less ALI induced by LPS	Yin et al., 2011
New Zealand white rabbit	–	Hemin	–	Neutrophils, macrophages and lung alveolar epithelial cells	More HO-1 leads to less ALI/ARDS induced by ventilation	An et al., 2011
Rat	Alveolar macrophages	Hemin	ZnPPIX	Alveolar macrophages	More HO-1 leads to more anti-inflammatory responses	Chen et al., 2012
Rat	Rat alveolar macrophage cells	Hemin	ZnPPIX	Lungs; alveolar macrophages	Lung oxidative stress injury reduced with HO-1 induction	Yu et al., 2016
Sprague Dawley rat	–	Hemin	–	Lungs	More HO-1 leads to less ALI induced by explosion	Chavko et al., 2008
Sprague Dawley rat	A549 cells	–	ZnPPIX	Lungs	More HO-1 leads to ALI induced by I/R	Wu et al., 2013

**Figure 1:**
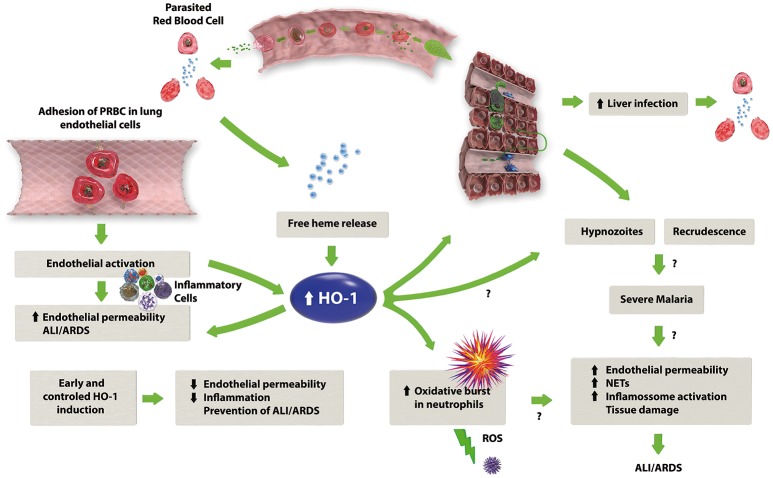
The proposed mechanism of HO-1 in the development of MA-ALI/ARDS. Malaria infection leads to the lysis of erythrocytes with consequent release of free heme, which in combination with the adhesion of infected erythrocytes to the endothelium, causes endothelial activation with subsequent release of pro-inflammatory cytokines and an increase in endothelial permeability. The release of free heme also upregulates HO-1 expression, which may have a dual role: on one hand, HO-1 activity results in the production of anti-inflammatory factors, such as CO and biliverdin, reducing the inflammation caused by malaria. On the other hand, HO-1 could stimulate the oxidative burst in neutrophils, leading to an increase in the inflammatory response.

As shown in the literature reviewed here, HO-1 has an important role in the development of severe malaria (Figure 1). It has also been shown that HO-1 can be modulated in a number of different ways to ameliorate the complications of malaria, particularly ALI/ARDS. This makes HO-1 a target for new drugs such as ALF492, which is efficient in reducing the severity of both ECM and MA-ALI/ARDS (Pena et al., 2012). Although most of the studies on HO-1 show that its induction is beneficial, (Table 4) the roles of this enzyme in diseases such as malaria must be determined in more detail to know which drugs, dosages and time points will be best in preventing ALI/ARDS.

### Final remarks and perspectives

The highly complex signaling and cellular arrangements that orchestrate lung injury represent serious challenges in developing new therapies for severe malaria complications, especially MA-ALI/ARDS.

Merely controlling malarial infections with anti-malarial drugs does not regulate pulmonary vascular permeability, since patients can develop ALI/ARDS after anti-malarial treatment is completed. Lung edema results in tissue hypoxia and organ dysfunction, leading to the high mortality observed among ALI/ARDS patients (Mohan et al., 2008).

The induction of HO-1 may be an important target in controlling pulmonary vascular permeability and inflammation (Pereira et al., 2016). However, we must not forget that malaria blood infection already increases HO-1 expression (Pamplona et al., 2007; Epiphanio et al., 2010; Pereira et al., 2016), which is associated with disease severity (Walther et al., 2012). The liver stage may coexist with the blood stage (HO-1 increase) in endemic areas where the infection rate is high and one individual could be infected multiple times in a short time frame (al-Yaman et al., 1997). As HO-1 has an anti-inflammatory effect, it promotes *Plasmodium* development and multiplication in the hepatocytes (Epiphanio et al., 2008), which increases parasite load and could contribute to the severity of the disease. In addition, it is completely unknown whether HO-1 expression might have some influence on hypnozoites or recrudescence cases, which increases the complexity in understanding the action of HO-1 in this context. Despite this, HO-1 induction was shown to be beneficial in preventing severe malaria syndromes, as cerebral malaria and MA-ALI/ARDS (Pamplona et al., 2007; Pena et al., 2012; Pereira et al., 2016). Additionally, HO-1 was increased during those syndromes not only in mice but also in humans (Takeda et al., 2005; Pamplona et al., 2007; Sambo et al., 2010; Cunnington et al., 2012; Walther et al., 2012; Pereira et al., 2016). Therefore, we hypothesize that high levels of HO-1 are a pausible biomarker of the severity of malaria. However, despite favoring the malaria parasite replication in the liver stage, HO-1 activity has significant role to prevent severe malarial syndromes, including MA-ALI/ARDS.

Understanding the HO-1 mechanisms at play during the different stages of *Plasmodium* infection is fundamentally important. Using new models, such as the human liver-chimeric FRG KO huHep mouse (Mikolajczak et al., 2015; in which it is possible to study the liver stage, the formation and activation of hypnozoites and the blood stage of infection at the same time) might be useful for discovering the true role of HO-1 in *Plasmodium* infections and its importance in severe malaria, especially MA-ALI/ARDS.

## Author contributions

MP and CM wrote the manuscript. SE wrote the manuscript and funded this work. All authors read and approved the final manuscript.

### Conflict of interest statement

The authors declare that the research was conducted in the absence of any commercial or financial relationships that could be construed as a potential conflict of interest.

## Abbreviations

ALI/ARDS: acute lung injury/acute respiratory distress syndrome
CCR: C-C chemokine receptor
CLP: cecal ligation and puncture
CO: carbon monoxide
CoPPIX: cobalt protoporphyrin IX
CXCL: chemokine (C-X-C motif) ligand
CXCR: chemokine (C-X-C motif) receptor
DHA: dihydroartemisinin
EBA: Evans blue dye albumin assay
ECM: experimental cerebral malaria
ELISA: enzyme-linked immunosorbent assay
ELISPOT: enzyme-linked immunospot
FACS: - fluorescence-activated cell sorting
FiO_2_: fraction of inspired oxygen
HbSAD: sickle hemoglobin
HO-1: heme oxygenase 1
HUVEC: human umbilical vein endothelial cells
ICAM-1: intercellular adhesion molecule 1
IFA: immunofluorescence assay
IFN: interferon
IHC: immunohistochemistry
IL: interleukin
KO: knockout
LPS: lipopolysaccharide
MA-ALI/ARDS: malaria-associated ALI/ARDS
MEC: murine vascular endothelial cells
MPO: myeloperoxidase
NET: - neutrophil extracellular trap
NK: natural killer
PaO_2_: arterial blood oxygen tension
PCR: polymerase chain reaction
PMN: polymorphonuclear
PRBC: parasitized red blood cell
RF: respiratory frequency
ROS: reactive oxygen species
siRNA: small interfering RNA
SnPP: tin protoporphyrin
TGF: transforming growth factor
TNF: tumor necrosis factor
TNFR1: tumor necrosis factor receptor 1
VEGF: vascular endothelial growth factor
XR: X ray
ZnPPIX: zinc protoporphyrin IX.
